# 

**Published:** 1909-03

**Authors:** 


### CONTENTS.

ORIGINAL COMMUNICATIONS—

Pneumonia. By James B. Baird, M. D., Atlanta, Ga. .. .. .. .. .. .. 615

Blood In the Urin—Some Cases. By Alfred L. Fowler, M. D., Atlanta, Ga. .. .. .. .. 622

Report of Three Cases of Empyema. By Chas. P. Ward, M. D., Atlanta, Ga. .. .. .. .. 631

Clinical Notes on Gonorrheal Cases Treated With Serum. By Melvin S. Rosenthal, M. D., Baltimore, Maryland .. .. .. .. .. .. 635

Acute Traumatic Tetanus Treated by Magnesium Sulphate. By. Aime Paul Heiecnk, Chicago, Ill. 636

Some Experiences In Surgery of the Appendix With Report of Cases. By R. R. Kime, Atlanta. 645

Sex Education. By Follen Cabot, M. D., New York .. .. .. .. .. .. 652

EDITORIALS .. .. .. .. .. .. 655

NEWS AND NOTES .. .. .. .. .. .. 660

BOOK REVIEWS .. .. .. .. .. .. 669

